# Photobiomodulation Therapy in the Management of Late Complications After Facial Filling

**DOI:** 10.7759/cureus.59513

**Published:** 2024-05-02

**Authors:** Maria da Glória B de Melo, Luciane H Azevedo, Luiz Fernando N Ruiz, Maristela M Lobo, Patrícia M de Freitas

**Affiliations:** 1 Prosthodontics, Instituto de Odontologia Glória de Melo, Brasília, BRA; 2 Faculty of Dentistry, Universidade de São Paulo, São Paulo, BRA; 3 Periodontics Division, Federal University of Goiás, Goiânia, BRA; 4 Facial Esthetics, São Leopoldo Mandic, São Paulo, BRA; 5 Special Laboratory of Lasers in Dentistry, Department of Operative Dentistry, School of Dentistry, Universidade de São Paulo, São Paulo, BRA

**Keywords:** dentofacial esthetics, laser, photobiomodulation therapy, orofacial harmonization, facial filler

## Abstract

The aim of the present study is to report a clinical case of a patient diagnosed with a late adverse reaction to the injection of filler material - persistent and intermittent delayed swelling (PIDS) - in which photobiomodulation therapy (PBMT) with low-power laser was used for edema reduction. This is an observational, descriptive, and retrospective work of a case report. The female patient, aged 73 years old, had undergone dermal filler six years before and complained of increased volume in the face region (glabellar region, labiomental sulcus, and nasolabial folds) and was submitted to ultrasound and anatomopathological analysis. PBMT using a low-power laser (660 nm and 808 nm, simultaneous irradiation, in contact, 2 J/point, 100 mW) proved to be effective for the non-invasive approach of late adverse reaction to dermal filler, such as PIDS, a common complication related to the use of dermal fillers.

## Introduction

Facial harmonization has been an area of ​​great advancement in aesthetic medicine, with a clear growth in procedures performed by dentists. Minimally invasive techniques represent a powerful tool for facial rejuvenation, and dermal fillers and botulinum toxin are popular modalities to restore and optimize facial proportions and harmony [1].

The objectives of the procedure with the use of fillers include the correction of age-related changes (bone resorption, stretching of supporting fibers, loss of adipose tissue, etc.) to the optimization of facial proportions [2], assuming that the proper use of these products requires knowledge of facial anatomy, analysis of morphology and facial aging, as well as the rheological characteristics of these products.

One of the most used products for facial fillers is hyaluronic acid, which can be available in different densities, related to purity, concentration, reticulation technology, and resistance to degradation of the material [3].

Despite the various aesthetic advantages resulting from the use of fillers, some intercurrences and complications may occur. The most commonly cited in the literature include opportunistic infections (of viral, fungal, or bacterial origin), inflammation, edema, erythema, necrosis, ischemia, pain, and/or impairment of neuromuscular activity [4]. In general, the most used treatment is medication [4]. However, during the last decades, there has been a growth in the use of light sources, especially low-power lasers, as promising alternative therapies, which are safe, effective, of relatively low complexity, and minimally invasive [5].

Photobiomodulation therapy (PBMT) with low-power lasers encompasses the therapeutic use of non-ionizing light, under red and infrared emission spectra. It is capable of promoting the modulation of physiological responses through cellular stimulation [5-8], which triggers non-cytotoxic and non-thermal reactions and results in modulation of the pain response (analgesia), tissue biomodulation (repair), and modulation of the inflammatory process.

Considering its high acceptance by patients, as it is a technique that requires relatively little clinical time and is of low complexity, safe, and painless [6], the use of laser in the treatment of intercurrences and complications in facial harmonization has been growing and contributing positively, mainly due to the effective reduction of pain and inflammation and, consequently, a reduction in the need for medication, offering greater comfort to the patient.

This article aims to report, for the first time, a clinical case of a patient diagnosed with a late adverse reaction to filler material, known as persistent and intermittent delayed swelling (PIDS), in which PBMT with a low-power laser was used to reduce edema.

## Case presentation

A 73-year-old female patient was referred to the dental office complaining of increased volume in the region of the face where she had facial filler injected six years before. According to the patient, in 2014, she underwent an aesthetic procedure with filler in the glabella region, labiomental sulcus (LMS), and nasolabial folds (NLF), with material that she was unable to report.

**Figure 1:**
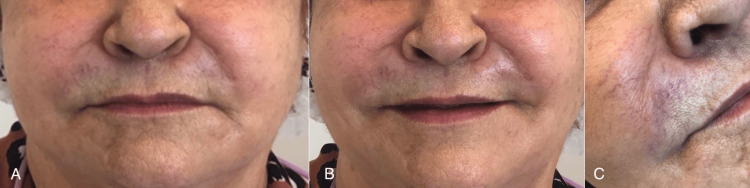
Initial photo. (A) Patient with lips at rest, (B) patient smiling, and (C) region of the nasolabial fold with increased volume (03/31/2021).

Six years after the filling procedure, the patient noticed the formation of irregular and hardened nodules on palpation (Figure 1) and a considerable increase in volume, making it difficult to chew (trauma to the buccal mucosa), limitation of mouth opening (difficulty in brushing and flossing), phonetics (more nasal voice), and facial discomfort. The patient also complained about the facial deformity, stating that she “would never stop wearing a mask”, an accessory that was mandatory during the COVID-19 pandemic.

**Figure 2:**
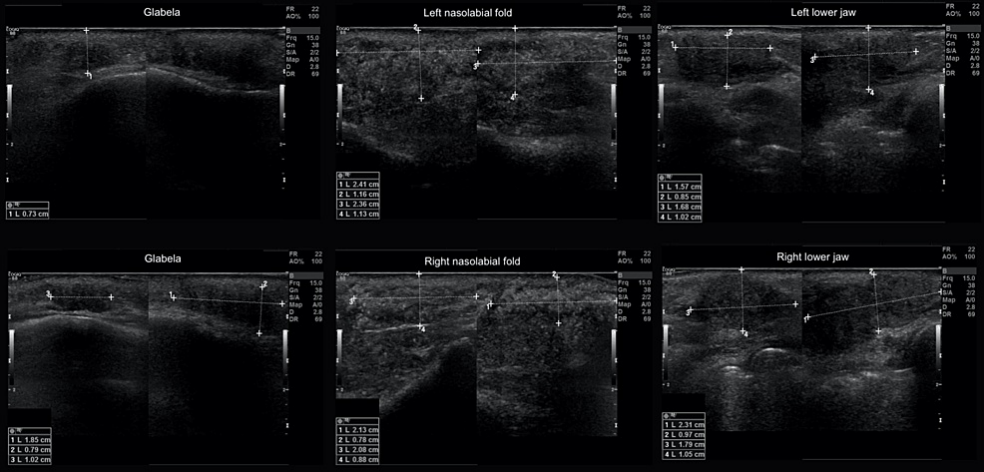
Ultrasound images identifying the dimensions and location of the dense, clinically palpable material.

On clinical examination, edema was observed in the region of the NLF and glabella and extra- and intraoral palpable rigid nodules. Upon palpation, the nodules in the region of the NLF were approximately 3.0 cm long and 1.0 cm wide, both on the right and left sides of the face. An ultrasound examination of the patient's face was requested to locate the materials. The examination was performed with a computerized device, i.e., a high-resolution multifrequency linear transducer (Figure 2). The presence of material was found in the subcutaneous planes (according to the report, suggestive of polymethylmethacrylate), distributed as follows: glabella region (measuring 1.8 x 0.7 x 1.0 cm, 0.7 cm away from the skin), right NLF (measuring 2.1 x 0.7 x 2.0 cm, 0.8 cm from the skin), left NLF (measuring 2.4 x 1.1 x 2.3 cm, 1.1 cm from the skin), right LMS (measuring 2.3 x 0.9 x 1.7 cm, 1.0 cm from the skin), and left LMS (measuring 1.5 x 0.8 x 1.6 cm, 1.0 cm away from the skin).

Based on the anamnesis, clinical examination, and complementary imaging examination (ultrasound), the diagnosis was of a late adverse reaction to the injection of the filler material, i.e., PIDS.

As a non-invasive therapeutic approach, PBMT with a low-power laser was chosen. The equipment used was a laser with emission in the red and infrared spectra (Therapy EC, DMC Equipamentos, São Carlos, SP, Brazil), with the parameters described in Table 1.

**Table 1:** Irradiation parameters used for PBMT. PBMT: photobiomodulation therapy.

Active medium	Diode laser (AsGaAl and InGaAlP)	
Wavelength	808 nm	660 nm
Energy/point	2 J	2 J
Beam area	0.098 cm²	0.098 cm²
Energy density	20.4 J/cm²	20.4 J/cm²
Time/point	20 s	20 s
Power	100 mW	100 mW
Power density	1.0 W/cm²	1.0 W/cm²
Number of points	12 (bilateral) extraoral and 5 (bilateral) intraoral	
Application mode	Continuous, in contact with the target surface	
Number of sessions	10	
Treatment time	4 weeks	

**Figure 3:**
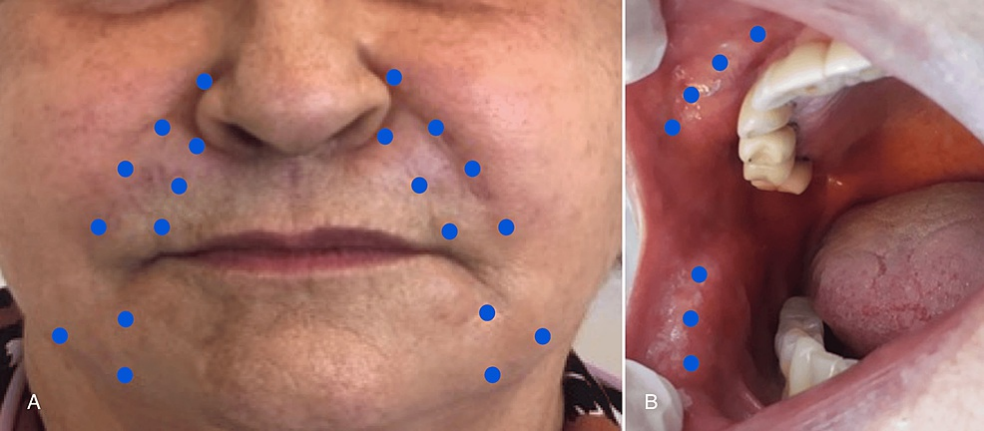
Application points used for PBMT. (A) Points considered for the extraoral irradiation. (B) Points considered for the intraoral irradiation, covering the densest region on palpation. PBMT: photobiomodulation therapy.

The protocol was performed at intervals of 48 hours (3x/week). A total of 10 sessions were performed, with points distributed in the region of the NLF, LMS, and glabella, and internally in the region of the jugal mucosa, where there were palpable nodules (Figure 3). From the first session, the application was made with the simultaneous emission of two wavelengths (red and infrared).

**Figure 4:**
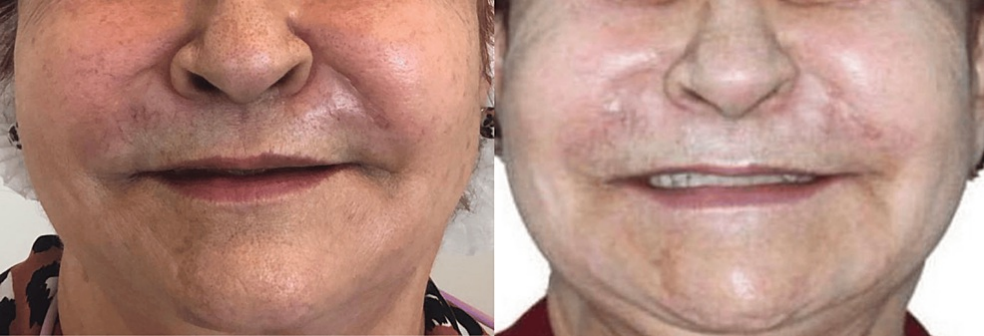
Before (left) and after (right) 10 sessions of PBMT. Reduction of swelling in the perioral region, allowing greater movement of the lips and exposure of the teeth. PBMT: photobiomodulation therapy.

After the 1st clinical session, the patient still had facial discomfort. After the 1st week of treatment, she reported greater ease in accessing posterior teeth with the brush and dental floss and greater comfort in the face. There was a visible reduction in edema, better visualization of the nodules (firm and irregular area, not painful to touch), and easier access of the laser tip to the intraoral irradiation points. After six sessions of PBMT, the patient reported, in addition to improved comfort, not traumatizing the buccal mucosa during mastication. Clinically, with the decrease in edema, the nodules became more evident, evolving positively, with a reduction in discomfort, in each subsequent session of PBMT. The patient's clinical evolution is illustrated in Figure 4.

**Figure 5:**
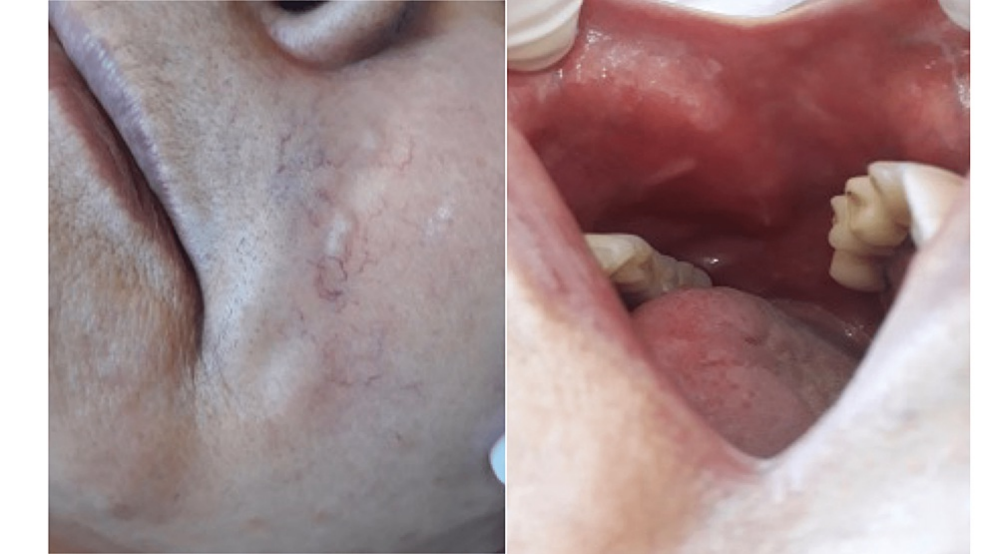
Presence of nodules, evidenced after edema reduction.

After the 9th session of PBMT, the patient had an episode of headache, which she associated with labyrinthitis, and used betahistine (8 mg). A small edema was observed all over the face. At the end of the 10 sessions of PBMT, the patient reported being satisfied with the therapy, with no discomfort on the face, normal chewing, and easy cleaning of the oral cavity, but complaining of nasal phonetics and discomfort with the nodules, as these were more evident after the reduction of edema on the face (Figure 5).

With the purpose of solving the aesthetic demand, generated by the evidence of the nodules, the patient was referred to a dental surgeon (L.F.N.) for evaluation and surgical removal of the dense, palpable tissue. Due to the high risk of permanent sequelae, including loss of vision, during the procedure for removing the material in the glabella region, surgery was performed only in the regions of the nasolabial folds and labiomental sulcus, with intra and extraoral accesses. The removed material was sent to the laboratory for anatomopathological analysis.

Anatomopathological examination

Macroscopic Aspects

The material received for examination consisted of several irregular, brownish, firm-elastic fragments, measuring 3.4 x 3.0 x 0.5 cm as a whole. All material was included for histological processing in hematoxylin-eosin (HE).

Microscopic Aspects

Histological sections revealed fragments of soft tissues showing marked fibrosis involving nodular, granulomatous inflammatory infiltrate, with numerous histiocytes and multinucleated giant cells of the foreign body type, which surround and phagocytize refringent globose structures of different sizes and optically negative round spaces. The presence of several lymphocytes was observed, with an absence of signs of malignancy. According to the examination report, the histopathological picture was compatible with a chronic granulomatous inflammatory process with a foreign body (heterologous material).

## Discussion

This clinical case shows a late response to a procedure using a filler material related to a delayed adverse reaction. These inflammatory reactions consist of challenging clinical conditions, which fall within the post-procedure complications, mainly of filler materials [9].

Although the etiology is still unknown, it is known that the immune system plays a prominent role with regard to late inflammatory reactions. In the present clinical case, the patient reported a medical history that may have influenced her immunological condition. Alijotas-Reig et al. (2018) [10] described that among the theories about the reason for delayed adverse reactions (as hypersensitivity) observed in patients who have dermal fillers, including triggers of viral infections, is that dermal fillers can act as adjuvants in the activation of T cells, increasing the antigen-specific immune response. A recent systematic review of the literature [9], which compiled data on immunological factors and bacterial contamination from 21 studies, showed that in situ bacterial contamination was hypothesized to cause these late inflammatory reactions, although its relationship with the immunological processes involved is unknown. The authors highlight that the presence of histiocytes, giant cells, and *Staphylococcus epidermidis* within biopsies is frequently associated with late inflammatory reactions. These data corroborate some of the findings of the anatomopathological analysis of the present case.

In general, the treatments most used to address these clinical conditions, such as the presence of late edema, are medication. However, more recently, the introduction of light sources, mainly low-power lasers, has been observed as a promising, effective, relatively low-complexity, and minimally invasive alternative [5].

De Freitas and Hamblin [6], in a publication that discusses the molecular, cellular, and tissue mechanisms of action, state that one of the most important chromophores absorbers of the light source in the red and infrared spectrum is cytochrome c oxidase (unit IV of the mitochondrial respiratory chain), which contains heme and copper centers, and absorbs laser light in the red and infrared spectrum. The main hypothesis is that photons dissociate inhibitory nitric oxide from the enzyme, leading to an increase in electron transport, mitochondrial membrane potential, and production of adenosine triphosphate (ATP) [6]. Another hypothesis concerns light-sensitive ion channels that can be activated allowing the entry of calcium (CA2+) into the cell. After the initial photon absorption events, several signaling pathways are activated through reactive oxygen species, adenosine monophosphate (AMP), nitric oxide (NO), and CA2+, leading to the activation of redox-sensitive transcription factors such as nuclear factor kappa B (NF-KB, a protein complex). These transcription factors can lead to increased expression of genes related to protein synthesis, cell migration and proliferation, anti-inflammatory signaling, anti-apoptotic proteins, and antioxidant enzymes [6,11].

Although the mechanisms of action of PBMT are not completely elucidated, it is believed that all the mechanisms discussed lead to a similar result, which is the modulation of the redox state of mitochondria (greater oxidation). Photoexcitation of the acceptor molecule (cytochrome C oxidase) sets cellular metabolism in motion through cascades of reactions called cellular signaling or retrograde mitochondrial signaling [12].

To date, there are no reports of the management of late edema following the injection of facial fillers, using the low-power laser. Almeida-Lopes and Lopes [13] discuss the use of PBMT and mention that the therapy is capable of reducing inflammation in a wide variety of clinical situations, either by reducing the formation of edema and the migration of inflammatory cells or by modulating the formation of inflammation-mediating substances (pro-inflammatory cytokines, such as tumor necrosis factor-alpha, interleukin I alpha, interleukin 6, prostaglandin E-2, and matrix metalloproteinases (MMPs)). An important observation is that inflammatory edema results from the action of histamine, followed by the effect of kinins and potentiated by the action of prostaglandins. Over 24 hours, the main factors responsible for edema being greater or lesser are prostaglandins. The action of drugs known as anti-inflammatory drugs is often based on reducing the local production of prostaglandins. Such prostaglandins will be important in the repair process and, therefore, the benefit of PBMT lies in the fact of modulating the local production of such prostaglandins, and not simply inhibiting their production [13].

Bernal Rodriguez et al. [14], in a study that reports a series of cases in which the association of PBMT with laser in the red (intraoral) and infrared (extraoral) spectrum was performed, proved the benefits of therapy in tissue repair in the suture region (red - intraoral) and edema/pain control (infrared - extraoral). This practice is supported by several other studies in the literature that use lasers with red emission for tissue repair [15-17] and in the infrared spectrum for control of edema and pain [17].

In the context of late inflammation, which can manifest as erythema, edema, and nodules [18], PBMT should be used for its control, promoting patient comfort. In some reports/series of cases, there was the involvement of an inflammatory process [14,19,20] and, although some studies have not described PBMT specifically for this purpose, the therapy contributed to the positive outcome of cases of bichectomy surgeries, ischemia, tissue repair, and edema, certainly by modulating local inflammation.

Even though it was not possible to determine which filler material was used and associate it with the late inflammatory process, it was possible to certify that, when dealing with edema and inflammatory processes on the face, PBMT with low-power laser proved to be effective in controlling and promoting of patient’s well-being, until the surgical procedure was performed.

## Conclusions

PBMT with low-power laser proved to be effective, with the described parameters (irradiated points, contact mode, simultaneous emission of 660 nm and 808 nm wavelengths, 2 J, 20 seconds per point, 100 mW), for the non-invasive approach to delayed hypersensitivity, such as PIDS, a common complication related to the use of dermal fillers.

The findings of the current case report are important in the scenario of facial aesthetic procedures as they present a low complexity, with low cost, effective, and clinically safe option for addressing possible clinical complications.
